# Supplementary figures and images for: Understanding experiments and research practices for reproducibility: an exploratory study
Source: PeerJ. 2021 Apr 21;9:e11140. doi: 10.7717/peerj.11140 (PMC8067906; doi:10.7717/peerj.11140)

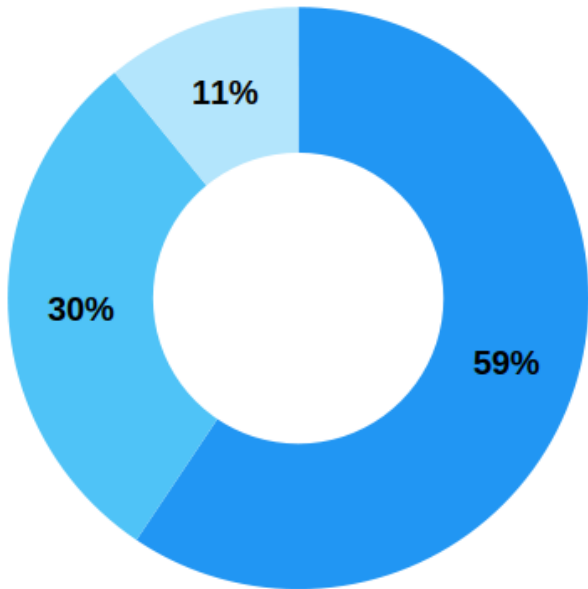

■ Yes  
■ No  
■ Other

Supplement: Figure S1 [file peerj-09-11140-s001.pdf]

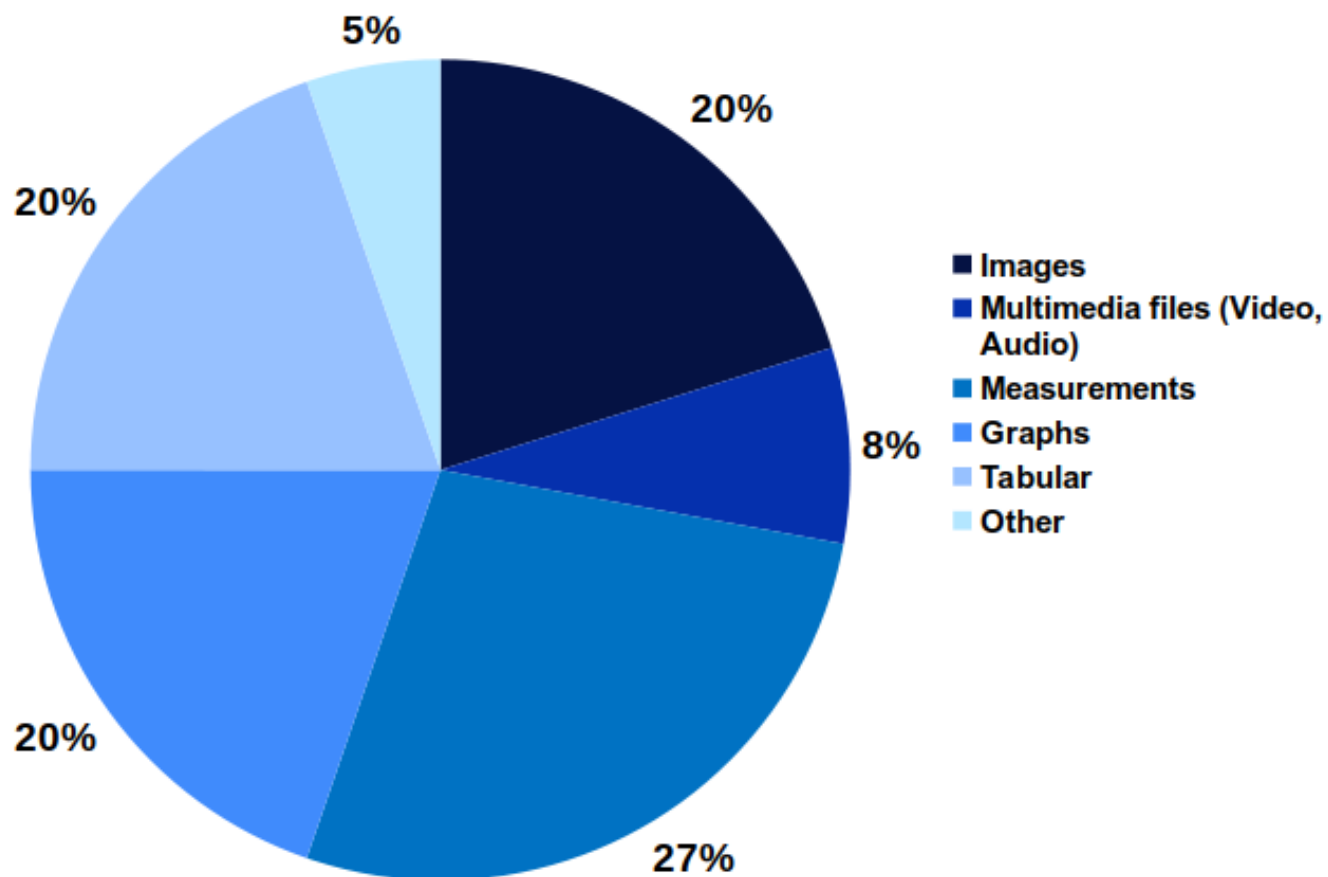

Supplement: Figure S2 [file peerj-09-11140-s002.pdf]

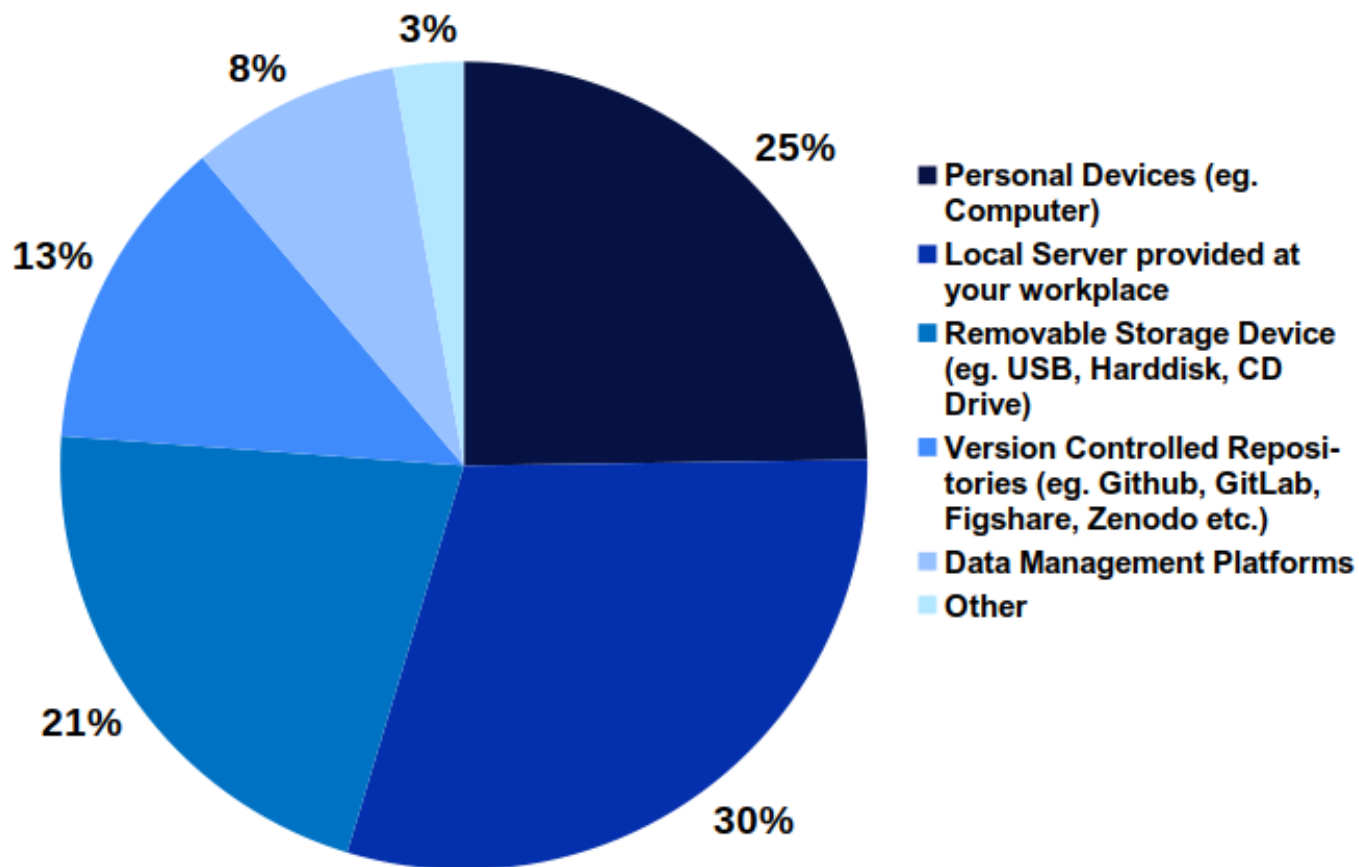

Supplement: Figure S3 [file peerj-09-11140-s003.pdf]

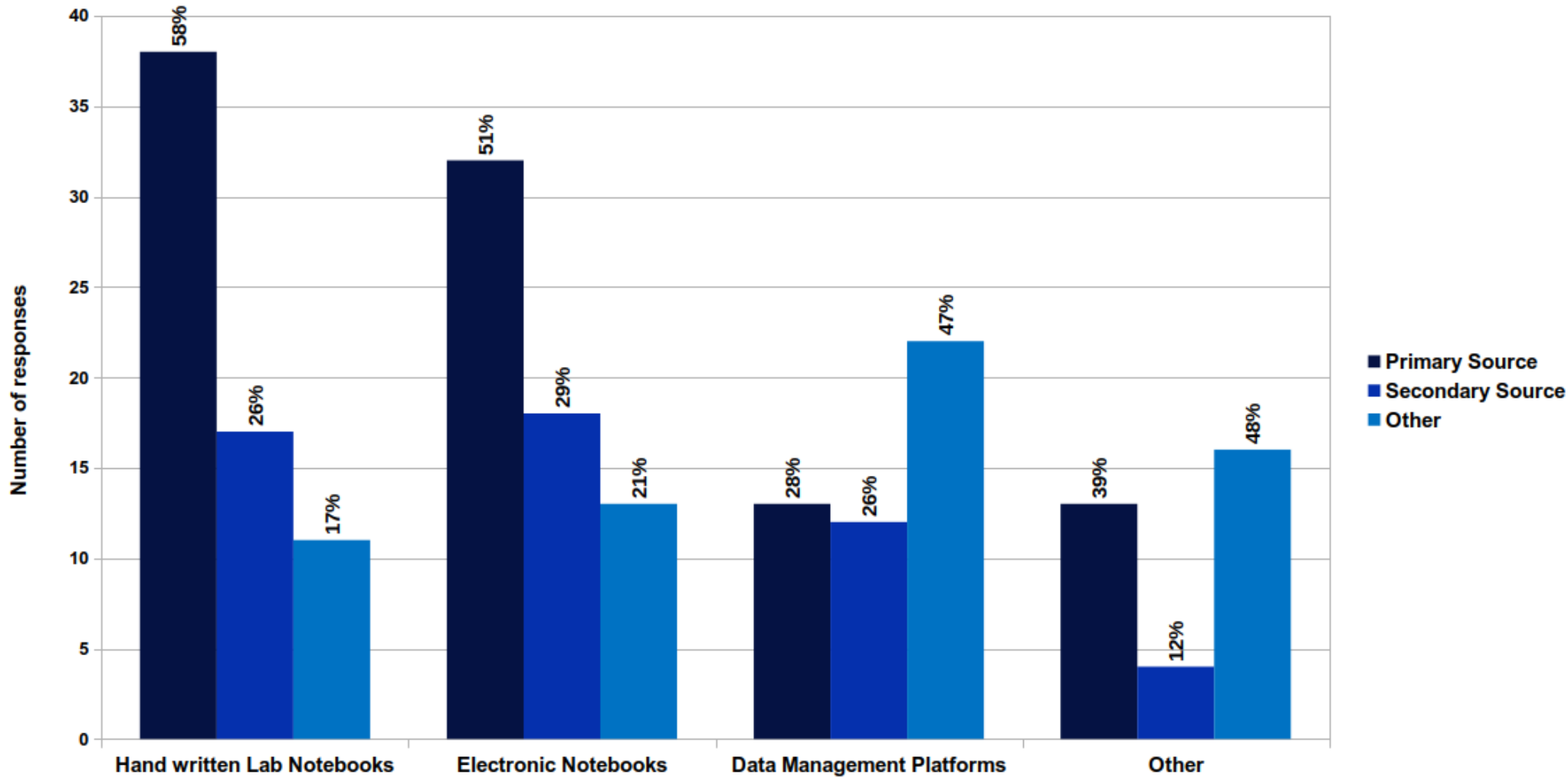

Supplement: Figure S4 [file peerj-09-11140-s004.pdf]
