# Supplementary material for: Understanding experiments and research practices for reproducibility: an exploratory study
Source: PeerJ. 2021 Apr 21;9:e11140. doi: 10.7717/peerj.11140 (PMC8067906; doi:10.7717/peerj.11140)
Supplement: Figure S5 [file peerj-09-11140-s005.pdf]

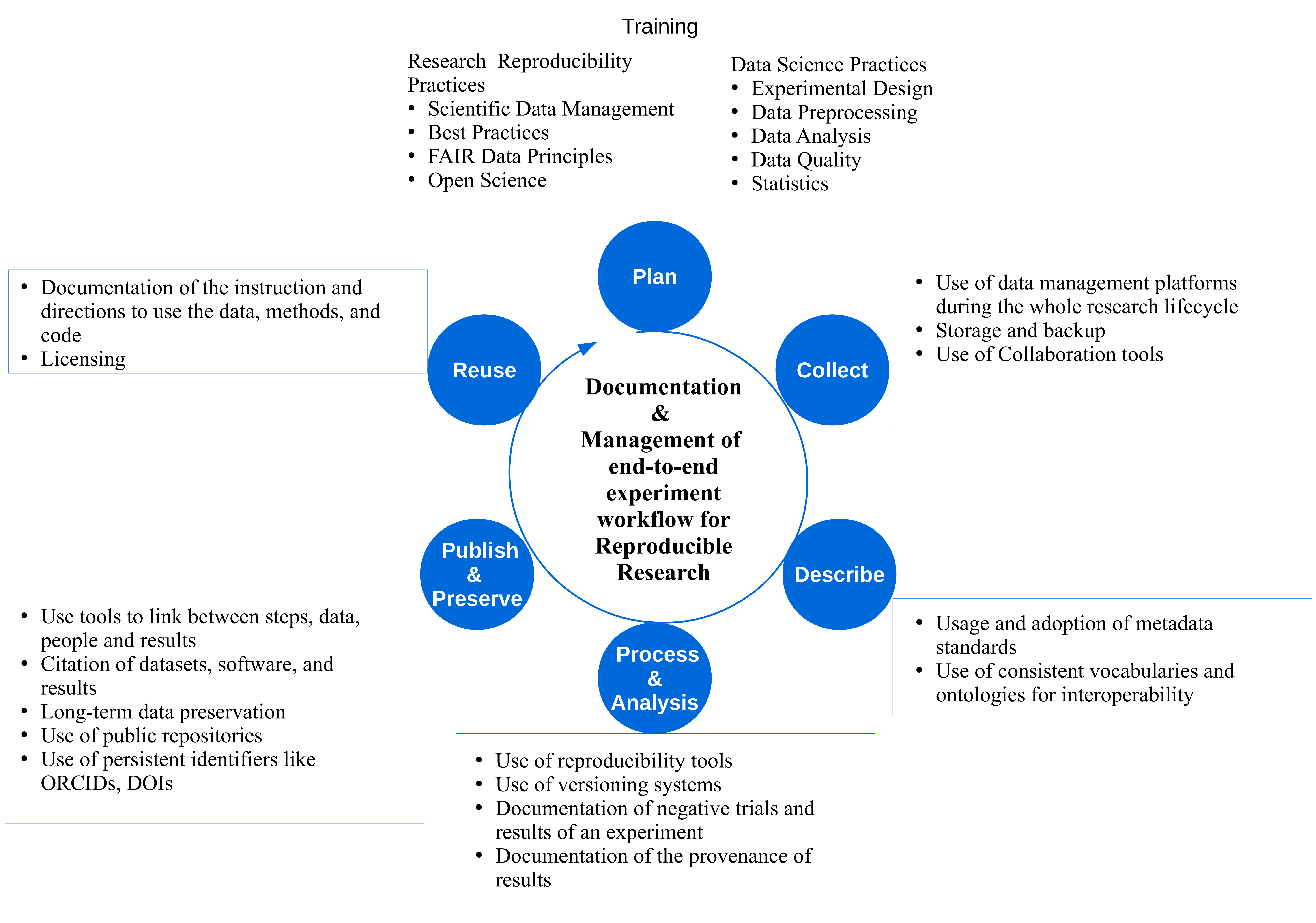
Training
Research Reproducibility Practices
• Scientific Data Management
• Best Practices
• FAIR Data Principles
• Open Science
Data Science Practices
• Experimental Design
• Data Preprocessing
• Data Analysis
• Data Quality
• Statistics
Plan
Collect
• Use of data management platforms during the whole research lifecycle
• Storage and backup
• Use of Collaboration tools
Describe
• Usage and adoption of metadata standards
• Use of consistent vocabularies and ontologies for interoperability
Process & Analysis
• Use of reproducibility tools
• Use of versioning systems
• Documentation of negative trials and results of an experiment
• Documentation of the provenance of results
Publish & Preserve
• Use tools to link between steps, data, people and results
• Citation of datasets, software, and results
• Long-term data preservation
• Use of public repositories
• Use of persistent identifiers like ORCIDs, DOIs
Reuse
• Documentation of the instruction and directions to use the data, methods, and code
• Licensing
Documentation & Management of end-to-end experiment workflow for Reproducible Research
