# Supplementary material for: Understanding experiments and research practices for reproducibility: an exploratory study
Source: PeerJ. 2021 Apr 21;9:e11140. doi: 10.7717/peerj.11140 (PMC8067906; doi:10.7717/peerj.11140)
Supplement: Table S1 [file peerj-09-11140-s006.pdf]

	Yes	No	Other	Total
**Current Position**				
PhD Student	20	7	0	27
PostDoc	13	4	1	18
Professor	2	7	4	13
Data Manager	4	3	1	8
Research Associate	3	3	1	7
Student	4	0	1	5
Junior Professor	2	1	1	4
Lecturer	0	1	0	1
Technical Assistant	1	0	0	1
Other	11	4	2	17

Table S1. Reproducibility crisis based on the participants grouped based on their position
