# Supplementary material for: Understanding experiments and research practices for reproducibility: an exploratory study
Source: PeerJ. 2021 Apr 21;9:e11140. doi: 10.7717/peerj.11140 (PMC8067906; doi:10.7717/peerj.11140)
Supplement: Table S2 [file peerj-09-11140-s007.pdf]

Area of Study	Yes	No	Other	Total
Computer Science	13	4	2	19
Biology(other)	10	5	2	17
Environmental Sciences	4	7	2	13
Molecular Biology	4	2	0	6
Neuroscience	4	2	0	6
Physics	4	0	0	4
Plant Sciences	2	1	0	3
Health Sciences	2	0	1	3
Cell Biology	2	0	0	2
MicroBiology	1	0	0	1
Chemistry	0	1	0	1
Other	14	8	4	26

Table S2. Reproducibility crisis in each field of the participants
