# Supplementary material for: Understanding experiments and research practices for reproducibility: an exploratory study
Source: PeerJ. 2021 Apr 21;9:e11140. doi: 10.7717/peerj.11140 (PMC8067906; doi:10.7717/peerj.11140)
Supplement: Table S3 [file peerj-09-11140-s008.pdf]

	Not Important At All	Little Importance	Average Importance	Very Important	Absolutely Essential	Not applicable
Raw Data	1%	9%	22%	30%	28%	10%
Processed Data	0%	4%	21%	33%	40%	3%
Negative Results	1%	1%	14%	47%	37%	0%
Measurements	0%	4%	21%	40%	31%	5%
Scripts/Code/Program	0%	6%	14%	30%	48%	2%
Image Annotations	1%	8%	18%	30%	30%	14%
Text Annotations	1%	11%	20%	26%	29%	13%
Experiment Materials	0%	3%	9%	31%	53%	5%
Instruments/Devices Used	0%	4%	11%	36%	45%	4%
Instrument Settings	0%	5%	9%	38%	42%	6%
Experiment Environment Conditions	0%	1%	15%	38%	38%	7%
Publications used	1%	2%	25%	42%	26%	4%
Names of people who are directly involved	3%	1%	23%	34%	36%	4%
Contacts of people who are directly involved	3%	4%	28%	34%	31%	1%
Roles of people who are directly involved	4%	18%	24%	31%	23%	1%
Names of people who are indirectly involved	15%	33%	30%	15%	5%	3%
Contacts of people who are indirectly involved	15%	33%	30%	13%	5%	4%
Roles of people who are indirectly involved	19%	34%	29%	10%	5%	4%
Date	4%	16%	25%	28%	22%	5%
Time	5%	22%	21%	27%	20%	5%
Duration	2%	11%	17%	41%	25%	4%
Location	10%	23%	16%	25%	21%	5%
Software Parameters	0%	2%	12%	37%	43%	5%
Software Version	1%	6%	14%	41%	36%	2%
Software License	7%	22%	30%	20%	17%	4%
Scripts/Code/Program	0%	4%	14%	35%	44%	4%
Laboratory Protocols	1%	1%	14%	41%	32%	11%
Methods	0%	1%	4%	39%	54%	3%
Activities/Steps	0%	2%	16%	51%	30%	1%
Order of Activities/Steps	1%	4%	18%	44%	33%	1%
Validation Methods	0%	1%	15%	49%	32%	2%
Quality Control Methods	0%	2%	21%	43%	30%	4%
Final Results	0%	3%	9%	33%	53%	3%
Intermediate Results	0%	17%	40%	32%	9%	3%

Table S3. To reproduce published experiment results, what is your opinion on sharing metadata on the 34 factors
